# Unilateral eyelid edema and mucosal involvement as the first presentations of Wegener granulomatosis

**DOI:** 10.22088/cjim.10.3.343

**Published:** 2019

**Authors:** Rana Rafiei, Narges Alizadeh, Kaveh Gharaei nejad, Behnam Rafiee, Sara Najirad

**Affiliations:** 1 Skin Research Center, Department of Dermatology, Razi Hospital, Guilan University of Medical Sciences, Rasht, Iran; 2 Department of Pathology, NYU Winthrop Hospital, 222 Station Plaza, N#620, Mineola, NY, 11501, USA; 3 Nassau University Medical Center, Department of Internal Medicine, 2201 Hempstead Turnpike, East Meadow, NY, 11554, 516-978-0158, USA

**Keywords:** Wegener’s granulomatosis, Oral lesions, Eyelid edema

## Abstract

**Background:**

Wegener granulomatosis or granulomatosis with polyangiitis is a pauci-immune small vessel vasculitis which is usually associated with anti-neutrophil cytoplasmic antibodies (ANCA) mainly in old men. This small vessel vasculitis is usually characterized by necrotizing granulomatous inflammation with multiorgan involvement. Kidneys could be involved as the main and life-threatening condition in Wegener granulomatosis. Oral or ocular lesions may occur as the first and uncommon presentations before internal organ involvement in these patients and could be misdiagnosed with other diseases.

**Case presentation:**

We present a 24-year-old man with erosions and ulcerations on palatal mucosa and a strawberry-like gingival hypertrophy associated with nasal congestion and epistaxis which two stated months ago. Also he had an episode of unilateral blepharitis and upper eyelid edema five months ago. Mucosal biopsy showed perivascular infiltrations of mainly neutrophils, some eosinophils and rare giant cells. He had elevated level of proteinase 3–ANCA or C-ANCA with microscopic hematuria without significant kidney involvement in kidney biopsy. Mucosal lesions and hematuria improved after two months of treatment with oral prednisolone.

**Conclusion:**

Unilateral eyelid edema and mucosal erosions in a young man could be the uncommon presentations of Wegener granulomatosis.

Granulomatosis with polyangiitis (GPA) or Wegener granulomatosis (WG) is a pauci-immune small vessel vasculitis mainly in old age. It is characterized mainly by necrotizing granulomatous inflammation, systemic necrotizing vasculitis, and segmental necrotizing glomerulonephritis which is usually associated with anti-neutrophil cytoplasmic antibodies (ANCA). Renal involvement is an important and life-threatening condition in WG (1, 2). GPA could present as mucosal or ocular lesions before systemic involvement so correct diagnosis may be delayed in these situations. Early diagnosis and management of these conditions could reduce mortality and morbidity of the disease (3-5). GPA is very rare in young adults (6). Herein we present a young man who had unilateral eye lid edema and mucosal involvement as the first presentations of GPA. 

## Case presentation

A 24-year-old man was referred to our outpatient clinic due to ulcerations of palatal mucosa, nasal congestion and epistaxis two months ago. He had an episode of arthralgia that started one week ago. 

**Figure 1:**
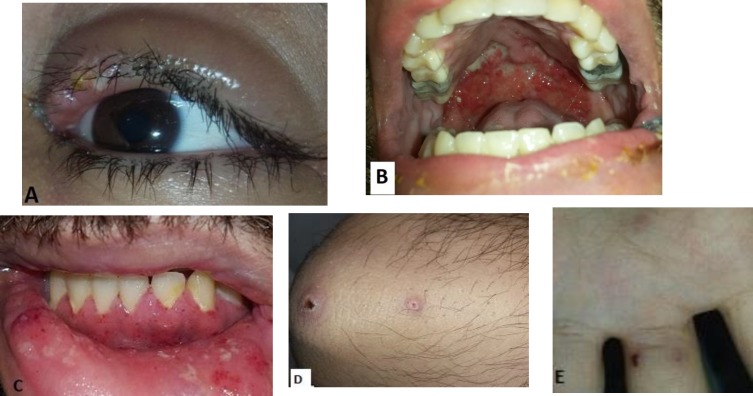
A- Unilateral upper eyelid edema and blepharitis with pustule formation, B- Erosive and ulcerative lesions on the hard palate ,C- Strawberry-like gingival hypertrophy, D-Ulcerative papulonodules on the right elbow, E- palmar purpuric papules are seen

Also, he had a history of unilateral blepharitis and upper eyelid edema five months ago (figure 1a). On physical examination, strawberry-like gingival and labial hypertrophy, erosive and ulcerative lesions on the hard palate, a few ulcerative papulonodules on the right elbow, palmar purpuric papules, and nasal crusted hemorrhagic debris were detected (figure 1b,c,d,e) .

**Figure 2:**
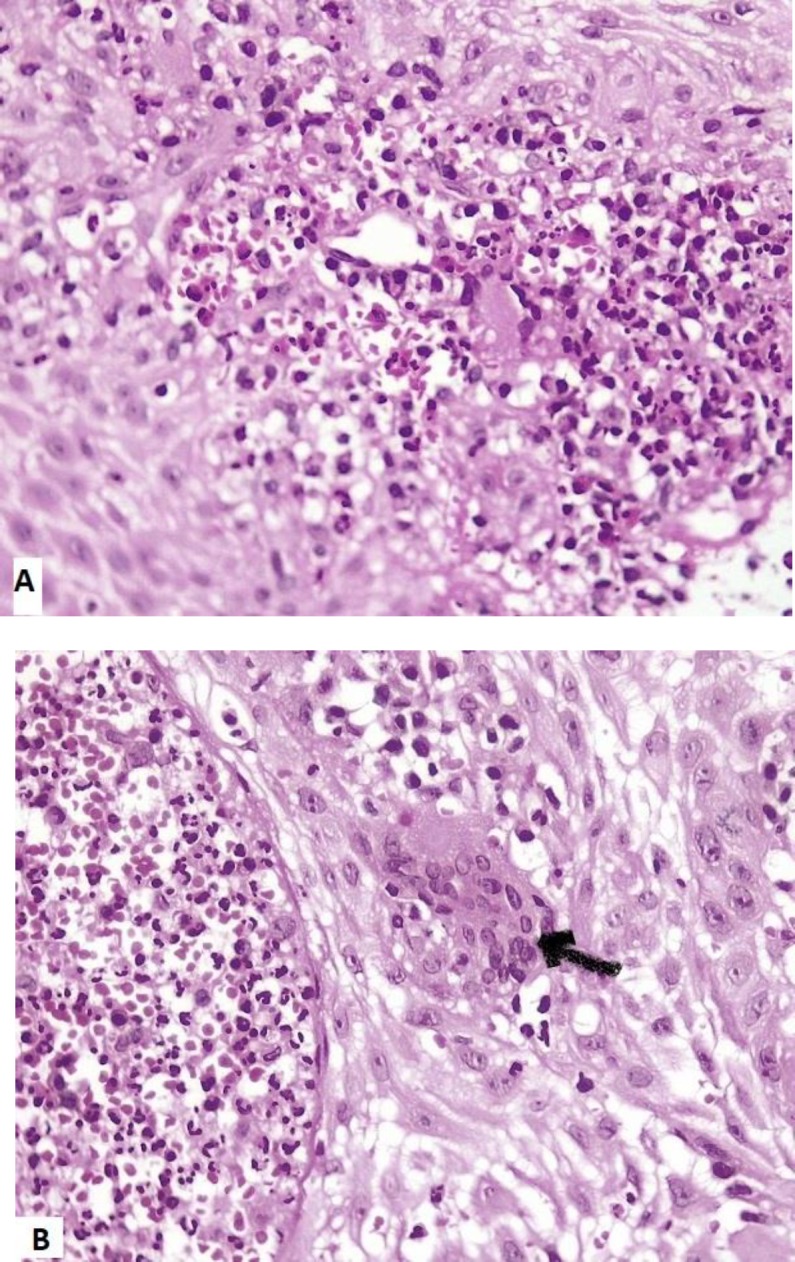
A- Foci of vasculitis involving small-sized vessels as well as foci of red blood cells extravasation are evident. B-Diffuse dermal infiltrations of neutrophils admixed with large number of plasma cells and eosinophils as well as some lymphocytes, histiocytes and multinucleated giant (pointed by an arrow) are seen. (Hematoxylin-eosin, original magnification: × 400)

Eyelid edema had been resolved with a topical steroid. There was no history of constitutional symptoms. Past medical history and drug history were unremarkable. Laboratory work-ups were as follows: hemoglobin 11.8 g/dL, leukocyte count 8460 /µL with 5.6% eosinophils, erythrocyte sedimentation rate 47 mm/h, C-reactive protein (CRP) 2+ , blood urea nitrogen (BUN) 9 mg/dl (reference, 7-21), creatinin 1 mg/dl (reference, 0.7-1.4), serum immunoglobulin E (IgE) 2300 IU/mL (reference, up to 200), anti proteinase 3 (C-ANCA) 67.5 Au/mL (reference, positive: >18), hematuria [many red blood cells (RBCs) in urine analysis], dysmorphic RBCs in urine sediment, cloxacillin- sensitive staphylococcus aureus growth in nasal culture. Anti-myeloperoxidase (anti MPO or P-ANCA), anti-nuclear antibody, rheumatoid factor levels, liver and thyroid function tests were within normal limit. Stool exam and culture and labial Tzanck smear had no specific findings. Viral serology for human immunodeficiency virus, hepatitis B or C virus was negative. Labial and nasal mucosal biopsy showed focally ulcerated epithelium with marked spongiosis and irregular acanthosis associated with diffuse dense dermal infiltration of inflammatory cells mainly composed of neutrophils, admixed with large number of plasma cells and eosinophils as well as some lymphocytes, histiocytes and few multinucleated giant cells which formed vague granuloma. In addition, foci of vasculitis involving small-sized vessels as well as foci of RBC extravasation were evident (figure 2a, b). Direct immunofluorescence study was negative. Also previous eyelid biopsy had been done by an ophthalmologist showed the same pathologic findings without any giant cell formation. Renal biopsy under ultrasound guide was done due to significant hematuria. Serial cortical renal tissue sections showed 23 glomeruli. All glomeruli had fairly normal appearance, except for mild mesangial hypercellularity in 2-3 glomeruli. Basement membranes were thin and regular without spikes and hole in PAS & Jones stains. There was no endocapillary proliferation, crescent formation or fibrinoid necrosis. Some tubules contained RBCs and rare RBC casts. Interstitium and vessels showed no inflammation. Direct immunofluorescence study was negative. Electron microscopic evaluation was not available. Chest and orbital computed tomography (CT) imaging and echocardiography were normal and paranasal sinuses CT scan showed mild mucosal thickening of maxillary sinuses. According to these findings, the diagnosis of GPA without significant renal involvement was proposed.

All mucosal and skin lesions and hematuria significantly improved using oral prednisolone 60 mg daily tapere dose during two months. Topical mupirocin and oral cloxacillin were prescribed for nasal carriage of staphylococcus aureus. Adjuvant therapy was not recommended for him after nephrologist consultation had been done due to non-significant involvement of kidney based on the biopsy. Mild microscopic hematuria was sustained after four months follow-up without proteinuria, dysmorphic RBCs or changing in BUN and creatinine levels.

## Discussion

GPA is a rare pauci-immune small vessel vasculitis more commonly occurs in Caucasians without sex predilection. GPA is usually diagnosed between 5th to 7th decades of life but may affect patients at any age and has been rarely reported in young adults or children (1, 2, 6). Probably some infections such as staphylococcus aureus in a genetically predisposed patient result in T-cell dependent reactions inducing pro-inflammatory cytokines production. These cytokines express proteinase 3 (PR3) on activated neutrophils which is a target for antineutrophil cytoplasmatic antibodies called C-ANCA or anti-PR3, then neutrophils degranulate and tissue injury occurs, although genetic susceptibility is required for pathogenic effects of PR3-ANCA in mice models. Both cell-mediated and humoral immune systems are involved in GPA which results in granulomatous and pauci-immune small vessel vasculitis features respectively. Staphylococcus aureus antigen seems to have a super-antigenic role in GPA and could result in recurrent attacks, so any active lesions or nasal carriage should be treated similar to our patient (1, 2).

Clinical presentations of GPA are variable. Orofacial involvement has been reported in 15 % of cases (6). Oral lesions have been detected in 10–62 % of them and as the first manifestation in 5%-6% of cases. These lesions present characteristically as erosive, ulcerative or nodular lesions associated with a pebbly or strawberry-like gingival hypertrophy due to superimposed purpuric dots. Histopathological criteria of GPA include vasculitis, granulomatous inflammation, multinucleated giant cells, and necrosis, but in gingival lesions histopathological findings are usually less specific, as follows: pseudoepitheliomatous hyperplasia, acute or chronic inflammation with multinucleated giant cell formation which in association with typical clinical findings could be diagnostic (3, 5, 6).

 Ocular involvement has been reported in 30%-50% of patients which involves different ocular parts such as orbit, eyelid, conjunctiva, lachrymal system, sclera, uvea and retina. Eyelid involvement may present as edema, trichiasis, entropion, and xanthelasma. Surprisingly orbital masses may present as an atypical eyelid edema in these patients (4). Cutaneous lesions in GPA are non-specific and include purpura, pyoderma gangrenosum-like ulcerations, acneiform papules and pustules, folliculitis, cutaneous ulcers and gangrene but ulcerative papulonodular lesions on the elbow are characteristic. Nasal and paranasal sinus involvement in GPA could present as follows: recurrent and persistent bloody nasal discharges, crusting ulcers, granulomatous lesions, nasal bridge collapse or paranasal sinus inflammation and tenderness (1, 2). 

Laboratory findings in GPA patients include: leukocytosis may be associated with eosinophilia, anemia, thrombocytosis, elevated ESR and CRP (ESR better than CRP correlates with disease activity). Most patients with GPA are ANCA positive, usually PR3-ANCA positive or less commonly myeloperoxidase (MPO-ANCA) positive. Women are more likely to be MPO-ANCA positive. All immunoglobulin levels especially IgE could be elevated in GPA similar to our patient (8). 

GPA could involve internal organs especially lungs and kidneys which have prognostic effects (1, 2, 7). Some patients with GPA develop end-stage renal disease in spite of treatment with corticosteroids and cyclophosphamide. Persistent hematuria despite clinical remission of GPA could be explained with chronic glomerular injury or low-grade active renal disease, but continuing immunosuppressive therapy in these cases will be associated with a higher risk of infection and malignancy. Renal re-biopsy and electron microscopic evaluation have been proposed as the gold standard method to evaluate the activity of kidney disease in patients with persistent hematuria especially associated with proteinuria (7). Our patient did not give consent for renal re-biopsy due to significant clinical improvement. We present this case because GPA is a rare type of small vessel vasculitis in young age, especially if it presents as mucosal involvement at the onset. Delayed or misdiagnosis of GPA is common and unfortunately survival rate of untreated cases after one year is only about 18% (3). 

In conclusion we should think about GPA in patients with oral lesions and eyelid edema, because these features may remain localized for a long period of time before progression to multiple-organ failure and death.
